# Pre_GI: a global map of ontological links between horizontally transferred genomic islands in bacterial and archaeal genomes

**DOI:** 10.1093/database/bav058

**Published:** 2015-06-16

**Authors:** Rian Pierneef, Louis Cronje, Oliver Bezuidt, Oleg N. Reva

**Affiliations:** Bioinformatics and Computational Biology Unit, Department of Biochemistry, University of Pretoria, Pretoria, Gauteng 0002, South Africa

## Abstract

The Predicted Genomic Islands database (Pre_GI) is a comprehensive repository of prokaryotic genomic islands (islands, GIs) freely accessible at http://pregi.bi.up.ac.za/index.php . Pre_GI, Version 2015, catalogues 26 744 islands identified in 2407 bacterial/archaeal chromosomes and plasmids. It provides an easy-to-use interface which allows users the ability to query against the database with a variety of fields, parameters and associations. Pre_GI is constructed to be a web-resource for the analysis of ontological roads between islands and cartographic analysis of the global fluxes of mobile genetic elements through bacterial and archaeal taxonomic borders. Comparison of newly identified islands against Pre_GI presents an alternative avenue to identify their ontology, origin and relative time of acquisition. Pre_GI aims to aid research on horizontal transfer events and materials through providing data and tools for holistic investigation of migration of genes through ecological niches and taxonomic boundaries.

**Database URL:** http://pregi.bi.up.ac.za/index.php , Version 2015

## Introduction

Insertions of mobile genetic elements (MGEs) vernacularly termed genomic islands (islands, GIs), which arise from various origins by means of horizontal transfer (HT), are integral in the evolution of bacteria. HT, defined as the non-genealogical transmission of genetic material from one organism to another, is considered as an evolutionary event which allows bacteria to rapidly adapt to fluctuating environmental and ecological pressures ( 1 , 2 ). The presence of islands in prokaryotic genomes is known, with the effect of these insertions significant and widespread ( 2 , 3 ). The level of persistence and fixation of these horizontally transferred genes suggest that a selective advantage was conferred on recipient organisms ( 4 ). HT has been linked to an organism’s lifestyle with events following different classification systems ranging from acquisitions of new genes, paralogs from existing genes and orthologous gene displacements from other lineages ( 3 ) generally complicating the phylogeny of bacterial species. Recent pan-genomics studies strongly suggest that microbial genomes are continuously sampling and/or shuffling their genomic information, rather than undergoing slow, progressive changes ( 5 ). The latter is noticeable and in support of the new uprising drug resistant and pathogenic strains ( 6 ). Furthermore, a single HT event may alter the phenotype and virulence characteristics of a microbe by transforming it from a benign to pathogenic form, followed by further accumulation of virulence determinants. This conforms to the hypothesis of genetic capitalism, which states that a successful integration and selection of foreign genetic elements increases the number of possible genetic transfer events in the future ( 7 ).

Phylogenetic and parametric methods, which are widely used in island prediction, utilize comparative genomics and sequence composition signatures ( 8 ). Oligonucleotide usage (OU) frequencies have been shown to constitute microbial genomic signatures ( 9–11 ). As such these were recognized as being practically important and considered to be a parametric method of island identification. These approaches are based on an assumption that alternative OU biases in a genomic locus may indicate its horizontal acquisition ( 12 ). However, the approach was criticized at the onset as not everything that might be distinguished in a genome by compositional alterations may be deemed foreign ( 13 ). Specificity and sensitivity of the method were improved in latter implementations, particularly in the SeqWord Genomic Island Sniffer (SWGIS) program used for island identification for the Predicted Genomic Islands database (Pre_GI) ( 14 ).

It was reported that the acquisition of novel genes by HT in addition to the rest of the host genome indicates the importance of adaptive and auxiliary function provided by HT ( 15 ). An investigation of islands is complicated by a high frequency of mutations including gene gain and loss; and an extraordinary spectrum of varieties in terms of genetic organization and functionality ( 15 , 16 ). The functional annotation of protein encoding genes in islands allows for further investigations in terms of function and evolutionary impact of HT and the preferences in islands exchange between bacteria. Islands provide bacteria with functional properties such as: pathogenicity ( 17–19 ); tolerance towards antibiotics, heavy metal ions and oxidative stress ( 20–22 ); and they may also provide bacteria with traits that are of biotechnological importance ( 23–25 ). Elucidation of genes present in islands and the functional roles which they play in a variety of bacterial organisms is increasingly becoming of relevance ( 8 ). Such entities require a repository to improve the annotation, comparison and phylogenetic study of the newly identified islands through comparison against sets of the previously identified island associated genes.

The current databases that are of great use in bacteriological research include IslandViewer ( 8 ), PAIDB ( 26 ) and ACLAME ( 27 ); they all are constructed to address specific questions and challenges in island research. The IslandViewer database incorporates three island prediction methods to identify horizontally acquired genomic fragments of all types in sequenced bacterial genomes but does not allow for the identification of ontological links between them. PAIDB contains a collection of verified pathogenicity islands (PAIs), a class of islands known to contribute towards microbial virulence. ACLAME is a comprehensive web-resource which was created with the aim to reconstruct reticulation events in bacterial genomes. This latter constitutes a list of islands which are associated with prophages and plasmids.

Island ontology is literally defined as the study of being/existence of an island and the categories or groups that they reside in. The identification of an island and all associations is the first principle in the study of HT and MGE. The availability of considerable amounts of sequenced genomes enables the prediction of various islands in multiple different hosts. This enables the forming of categories of being by similarity or differences. Island ontology and research in newly sequenced bacterial/archaeal genomes is enhanced through comparison to known islands and categories of islands.

Pre_GI presented in this study aims to identify the current global position of islands and genes contained within these islands in view of the universal bacteria/archaeal community dynamically exchanging genetic data. Island ontology as a separate entity and as part of a larger group forms the foundation of this relational platform. The database bridges the boundaries of current island repositories by merging knowledge of mobilizing and directional movement of genes and vectors through ecological niches and taxonomic borders. The employment of OU pattern (OUP) comparison to reflect the history of islands in terms of genesis from specific organisms and the time elapsed from integration into current host chromosome is sustained by the successful implementation of this hypothesis in the GOHTAM Web-server ( 28 ). Pre_GI houses a vast amount of islands, including but not limited to pathogenetic islands, grouped by DNA compositional similarity, DNA sequence similarity, estimated time of origination and gene content. The database displays relevant information in an accessible and friendly format to allow users the ability to browse the current content and search against Pre_GI with novel islands to identify their position in the global space of HT. Pre_GI offers an addition to currently used databases of MGE to aid users in locating the history and position of HT events in the world of bacteria and archaea.

## Methods

### Island prediction

The SWGIS standalone program was used to facilitate a large-scale analysis of prokaryotic genomes to identify islands in sequences obtained from GenBank. This automated computational tool allows for the identification of horizontally transferred islands in bacterial and archaeal chromosome and plasmid DNA ( 14 ). The identified islands were stored in GenBank file format which were feasible and appropriate for populating Pre_GI. SWGIS is freely available for download at www.bi.up.ac.za/SeqWord/sniffer/index.html .

### Analysis of ontological links between islands

Ontology was inferred by analysis of sequence similarity, gene content and DNA compositional similarity shared by islands.

### Island compositional comparison

OUP calculations and comparison algorithms were introduced in our previously published work ( 12 , 29 ). OUP similarity between islands is determined by comparing the lists of constituent 4-mers ordered by oligonucleotide frequencies ( 12 ). One hundred percent OUP similarity between islands is indicated by both islands having the same ordered oligonucleotide lists. OUP similarity between islands was used to pronounce compositional similarity. In other calculations the distance (100% minus pattern similarity) between OUP was used. It was illustrated that levels above 75% OUP similarity propose a common ancestry of islands or at least their involvement in common reticulation events ( 6 ). This value was treated as the floor threshold for inclusion and any values below were discarded.

### Island sequence comparison

Sequence similarity for the initial set of islands was obtained with all-against-all BLASTN for the entire island nucleotide sequences. All-against-all BLASTP was performed for all coding sequences contained within all islands. Both comparisons were done with an e-value of 10 ^−^^6^ to ensure a true reflection of homology.

### Island clustering

Clustering of islands was performed by the Markov Clustering Algorithm (MCL) ( 30 , 31 ) using OUP similarity values between islands as relational scores. Initially OUP similarity values between islands of below 75% were used as a cut-off to ensure removal of random links. The influence of biased over-representation of islands from closely related species, e.g. *Escherichia coli* , was reduced by the implementation of a ceiling threshold of 85% OUP similarity to reduce the impact of duplicate islands on clustering results. Large clusters, more than 50 islands, were subclustered to ensure the production of distinct and significant subgroups.

### Island cluster/subcluster representatives

Island representatives for each cluster/subcluster were needed for implementation of a heuristic OUP similarity search through the database and also to facilitate the management and amendments of the database. Representatives for non-overlapping clusters/subclusters were designated as the nodes with the highest number of edges in the specific cluster/subcluster, i.e. the island with the maximum number of OUP similarity values between 75 and 85% to others in the cluster/subcluster. Large clusters required multiple representatives as diverse members of a cluster may not share any significant OUP similarity. Each cluster/subcluster was inspected to ensure that all of its members are associated with at least one representative by an OUP similarity of at least 75% to ensure an omnipresent set of island representatives.

### Proposed island donor–recipient relationships

The proposition of donor–recipient movement is grounded in the assumption that the process of amelioration alters the island nucleotide composition from the time of insertion to reconcile with that of the host in which it occurs, yet for an extended time after insertion an island may be traced back to its origin by preserving compositional homomorphism with the donor ( 14 , 32 ). GOHTAM implemented a similar approach of ascertaining the origin of MGE ( 28 ). This approach was applied in Pre_GI with the comparison of OUP similarity values calculated for homologous islands hosted by different organisms to predict donor–recipient relationships. Significant OUP differences of homologous islands to that of hosts would indicate possible donor–recipient relations. A high OUP similarity of both homologous islands to one host with a lower OUP similarity to the other indicates likelihood that the latter host is the recipient of a given island from the former, which contains homologous MGEs. However, the possibility of transient hosts/donors is not excluded and should be taken into account. The proposed movement from donor to recipient is furthermore dependant on the species sampling and sequence availability.

### Pre_GI future update

Due to the computational intensity and a large-scale OUP similarity comparison, updates of the database with newly identified islands and assignments to existing cluster/subcluster will be based on OUP similarity searches against island cluster/subcluster representatives. Novel islands shall be incorporated in the cluster/subcluster with which the highest OUP similarity to an island representative was found. BLASTN and BLASTP searches of the newly predicted islands and coding sequences contained in novel islands will be performed across the totality of the database, respectively, to ascertain homology. Updates will be identifiable by the version and date to provide a distinction between datasets.

## Results

Pre_GI, Version 2015, is a web-based database which forms part of the SeqWord project that is freely accessible from http://pregi.bi.up.ac.za/index.php . This interactive database aids in islands research and will be updated on a regular basis to ensure the inclusion of significant and novel data. The users are able to browse current islands or search and compare their newly predicted islands against Pre_GI records.

The driving force behind the creation of Pre_GI was the development of a web-based analytical resource to discover the ontological relationships between bacterial MGE and reconstruct the global fluxes of genetic vectors through taxonomic barriers. Bearing this in mind, Pre_GI interface includes various diagnostic tools for the comparison of newly identified and presently residing islands and the reconstruction of possible donor–recipient movements between bacterial species.

The holistic aim of the database was to incorporate not only identified islands but all possible sequence and compositional comparisons of these islands and thereby offering users the opportunity to inspect ontological relationships. Having all this information at the user’s fingertips allows for rapid deconstruction of novel island relationships and ontology by means of sequence and composition-based comparison.

### Identification of Islands and overlaps with IslandViewer and PAIDB

The basic principle behind the SWGIS algorithm is to superimpose the values of several statistical parameters calculated for a sliding window that allows identification of loci with an alternative OUP and distinguishing between the different categories of these genomic fragments, such as giant genes with multiple repeats, *rrn* operons, clusters of genes for ribosomal proteins and highly expressed genes ( 12 ). Particularly, islands are identified by an alternative OU (increase *D* parameter) with lower internally normalized OU variance ( *RV* ) and an increase in globally normalized OU variance ( *GRV* ). The latter two parameters were combined into a parameter *V* = *GRV* / *RV* , the value of *V* stays closer to 1 in the core genome and significantly increases in loci covered by an island. Detail regarding calculation of these parameters was described in our previous publications ( 6 , 12 , 14 , 29 ). These parameters are calculated in SWGIS for genomic loci by the use of a sliding window approach, whereby values of genomic fragments of 8 kb with a 2 kb step are compared with the tetranucleotide usage values calculated for the whole genome. If the program recognizes a statistically reliable increase in the local distance *D* accompanied by a significant increase of *V* , the window shifts several steps back and repeats the analysis, this time with steps of 0.2 kb to identify exact borders of the insert. An empirical analysis was performed to generate optimal parametric threshold values to be used for *D* and *V* .

**Figure 1.**
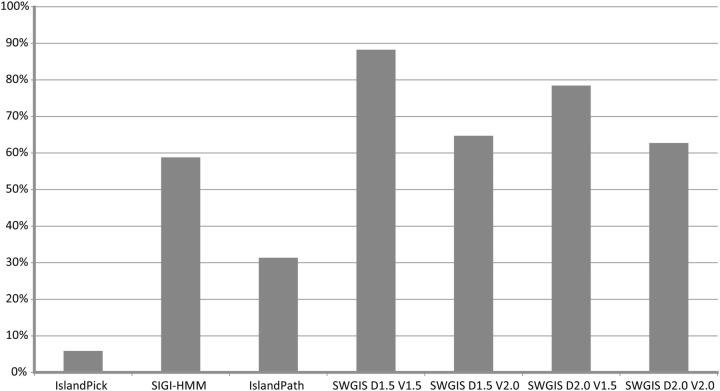
Percentage of re-identification of 51 known islands from PAIDB by IslandViewer tools and SWGIS with different threshold parameters.

The parametric measures for SWGIS were optimized to attain better predictions through the re-identification of known islands from PAIDB ( 26 ), which were used as training data. The SWGIS optimization and re-identification analysis was carried out on 51 PAIs possessed by 24 microorganisms. The latter was conducted in comparison to the IslandViewer programs comprising IslandPick, SIGI-HMM and IslandPath ( 8 ). From these comparisons the calculations for false negative rates (FNR) were determined. FNR in this instance is defined as the percentage of the known islands that were overlooked by either of the programs used in the study. SWGIS was run four times with different combinations of *D* and *V* : [ *D* :1.5; *V* :1.5]; [ *D* :2.0; *V* :2.0]; [ *D* :1.5; *V* :2.0] and [ *D* :2.0; *V* :1.5]. Results are shown in Figure 1. From the comparison of results attained from all programs, SWGIS outperformed individual IslandViewer methods even when the most stringent threshold values [ *D* :2.0; *V* :2.0] were set. Jointly the IslandViewer programs identified 69% of the 51 PAIs while SWGIS identified 88% with [ *D* :1.5; *V* :1.5], 78% with [ *D* :2.0; *V* :1.5], 65% with [ *D* :1.5; *V* :2.0] and 63% with [ *D* :2.0; *V* :2.0]. All PAIs predicted by the IslandViewer programs except for 2, which were only predicted by IslandPath, were also predicted by SWGIS [ *D* :1.5; *V* :1.5]. Four PAIs were not identified by any of the four methods. SWGIS also identified islands which were overlooked by other methods which poses the question of false positive predictions. It cannot be excluded that some of the islands, which were not predicted by other programs, were false positives. This is not easy to prove as there are no formal methods to prove that a given genomic fragment was not acquired by HT. To validate the predictions, an additional search for genes associated with MGE, e.g. ‘integrase’, ‘phage’, ‘IS-element’, was done and the results reflected on the Pre_GI island browser pages. However, an absence of these genes does not prove a false positive prediction due to inappropriate annotation, island fragmentation and other reasons. In previously published work ( 14 ) it was observed that in a sample of 1252 true positive islands only 56% of them contained the key words for mobile genes, which are termed ‘key positives’. From this observation the amount of true positives among unconfirmed islands was roughly estimated as ‘Number of unconfirmed but key positive islands’ × 100/56, with the other fraction of unconfirmed islands considered as false positives. By default the thresholds *D* and *V* were set in SWGIS to 1.7 and 1.5, respectively, that ensured an optimal sensitivity/specificity ratio. SWGIS was used in this study for a semi-automatic search for islands in multiple GenBank files of bacterial and archaeal chromosomes and plasmids, which were obtained from NCBI. The ‘Use BLASTn’ option is invoked by default in the standalone SWGIS program to enable BLASTN searches against an incorporated database of 16S rRNA sequences to identify the presence of *rrn* clusters in candidate islands. Candidates were marked as suspicious if they contained these clusters, as *rrn* genes are known to be resistant for horizontal exchange but resemble islands due to the alterations in OUP ( 12 ). The predicted islands were infused in Pre_GI.

## Pre_GI content

At the time of writing of this paper Pre_GI, Version 2015, contained 26 744 islands found in 2407 bacterial and archaeal chromosomes and plasmids. All islands were compared against each other to identify OUP, nucleotide and encoded protein sequence similarities. In our previously published work ( 6 ) it was demonstrated that islands with the level of OUP similarity above 75% very likely have a common ancestry and thus more frequently share homolog sequences. Common ancestry means that islands originated from closely related MGE such as phages or plasmids, or resulted from a direct transfer of genes from a donor organism to a recipient organism.

It should be reiterated that SWGIS is a composition-based island identifier and quite often ribosomal proteins characterized by an alternative OU are recognized by this program as islands. However, recent publications showed that horizontal ribosomal protein transfer cannot be excluded ( 33 ). Therefore, all islands containing ribosomal proteins or RNA-related elements are marked with an asterisk (*) in Pre_GI to ensure an unbiased and informative database.

The novelty of this database is that in addition to the information on location and genetic content of identified islands it provides data on ontological links between them which are either pre-calculated or generated on the fly as per user request. This functionality of the database allows users to address a variety of research problems including tracking down the circulation of related islands sharing specific virulence or catabolic genes; and identifying bacterial species harbouring relict versions of these islands and those organisms which acquired these genes recently.

To address these research questions, the Pre_GI web-interface was equipped with functional links to retrieve the requested data from the database and/or to allow users to upload newly identified islands to search for similarities through the database. Below we explain the essential functions and their applicability. More helpful information and several case study analysis are available at http://pregi.bi.up.ac.za/pre_gi_help.php .

### Pre_GI browse

Users are able to search the database and filter records to view islands of interest by using NCBI accession numbers, strain description, taxonomic data and general information on habitat and location of isolation. Criteria of filtering of records may be used in parallel. Another practical option is to search for islands by gene annotation key words. For example, a list of all islands containing efflux proteins or beta-lactamases may be retrieved by using this function. Names of bacterial organisms in the filtered list are hyperlinked to corresponding island browser pages that visualize all detected islands of the selected replicon in a SVG graph. Each visualized island contains information on genomic location, compositional similarity, sequence similarity and OU statistical parameters displayed in the form of a table below the graph. Islands with gene annotations containing description(s) that confer with HT event are indicated by Key Word Confirmation. Overlaps with IslandViewer and PAIDB are available as confirmations that the islands were predicted by several alternative approaches and as links to relevant databases. All genes contained in the island may be investigated by means of the hyperlinked start location of the island in the table. This table includes coordinates, BLASTP hits for the specific gene, gene annotation and a functionality to search the database for genes in other islands with a similar annotation. Each island’s GenBank text file is available for download. Statistical OU parameters of a given island can be compared to the rest of the database to obtain islands identified with similar statistical OU parameters. BLAST high scored hits obtained from all-vs-all island sequence similarity searches can be accessed and visualized through the added options of BLASTN hyperlinks, which return pages with graphical visualization of BLAST alignments and access to BLAST output in text format. OUP similarity hits for a chosen island reaching above 75% are presented in OUP Neighbours and OUP MCL cluster/subcluster pages. These pages allow viewing individual islands identified in other organisms which share a significant compositional similarity with the selected query island and thus may share a common ancestry with it. Proposed donor–recipient interactions between the query and subject islands are also presented as it is explained in a following section.

### Pre_GI island lineage and information

The inclusion of bacterial host lineage and general information in combination with sequence and composition similarity provides a novel avenue in HT and MGE research. Holistic island investigation is aided by host habitat and isolation references to ensure a holistic view of island ontology and origin.

This added information may assist research in the understanding of HT by emphasizing the natural behaviour, location and characteristics of bacterial/archaeal hosts of islands.

The taxonomic information obtained from the NCBI for host organisms was used in the calculation of general taxonomical statistics for the database and clusters/subclusters. Statistics on the taxonomic tallies are available for the chosen cluster/subcluster. Taxonomy statistics could enhance research on the propensity of islands in certain taxonomical groups.

### Pre_GI proposed donor–recipient flux

**Figure 2.**
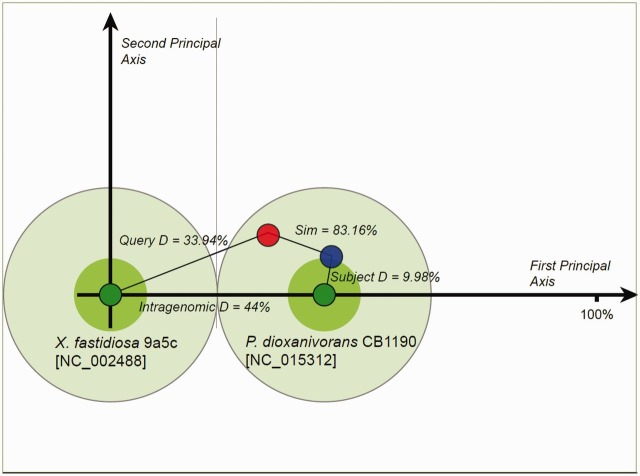
Proposed donor–recipient relationship by using LingvoCom 2D projection utility. Two dark green spots on the plot represent OUP of *X. fastidiosa* 9a5c (at centre point) and *P. dioxanivorans* CB1190 (on first principal axis) chromosomes. Light green circles depict 1/2 of the distance between patterns calculated for the chromosomes. The island of *X. fastidiosa* is shown as a red small circle and that of *P. dioxanivorans* as a blue circle. Islands were plotted along the second principal axis according to distance values between OUP of islands and host chromosomes.

Proposed donor–recipient indicators for homologous islands are presented in the OUP Neighbours, OUP MCL Cluster Neighbours and OUP MCL Sub Cluster Neighbours web-pages. This information may aid in detection of fluxes of MGE between bacterial/archaeal species. Probable direction of movement is indicated by arrows with green arrows depicting movement from the subject to the query and blue arrows depicting movement from query to subject. Red two-headed arrows signal that direction of movement is ambiguous. These pages allow for the retrieval of these relationships by specifying the direction of flux which is of interest. This analysis was conducted using LingvoCom, a collection of utilities which allow for the further analysis of islands in search for their potential donor genome. The tool is freely available for download from www.bi.up.ac.za/SeqWord/lingvocom/ . In the example demonstrated in Figure 2 , a large island located at 1 638 946–1 700 655 on the chromosome of *Xylella fastidiosa* 9a5c was considered. As indicated on the OUP Neighbours result page of Pre_GI, this island possibly originated from some *Pseudonocardia* as the strain *Pseudonocardia dioxanivorans* CB1190 contains a compositionally similar island [5 876 957–5 900 279]. The two islands from both organisms present a high OUP similarity to *P. dioxanivorans* CB1190 and lower similarity to *X. fastidiosa* 9a5c. It should be noted that this approach defines a possible direction of island exchange. It should not be excluded that there might be many intermediate bearers of this island. The proposed flow between donor–recipient hosts is also dependent on the current species sampling and availability of sequenced genomes and may change in future versions.

### Pre_GI annotation browser

All gene annotations may be searched and linked to display all similar gene annotations contained in the database. Sequences of islands and the genes they comprise are available for download in FASTA format. Annotations are linked to the QuickGO browser from EMBL-EBI ( http://www.ebi.ac.uk/QuickGO/ ) for gene ontology terms and annotations.

### Pre_GI search and analysis of novel Islands

Novel predicted islands may be queried against the database through locational, sequence and compositional similarity searches. This enables users to compare newly predicted islands against database records to obtain and investigate ontological links and similarities between islands. The ability to compare entire islands and not only genes enhances the functionality and applicability of the database to identify ontology and origin of islands. The submission to Pre_GI is possible in FASTA or GenBank format. To predict horizontally acquired islands in bacterial genomes the users may use IslandViewer ( 8 ) or any other relevant publicly available or in house programmes; however, we suggest the use of SWGIS. Prediction of the donor–recipient interactions between newly identified islands and those in Pre_GI is possible only when the predicted islands are submitted in GenBank files returned by SWGIS as these files contain additional necessary information.

### Pre_GI locational search

Existing genomes may be queried through coordinates to recognize if the region overlaps with islands present in Pre_GI. This may be used to indicate if the islands predicted by other methods overlap with SWGIS predictions, or to test if a gene of interest is contained in a predicted island. This application is available as ’search by location’ from the home page.

### Pre_GI sequence similarity search

**Figure 3.**
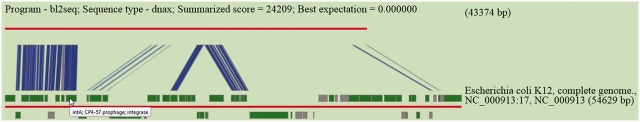
Pre_GI visualization of BLASTN comparison of the DNA sequence of a newly identified island against Pre_GI. The query sequence is depicted by the top red line and a hit subject sequence is represented by the bottom red line. Dark green boxes represent genes. Connecting blue strips between query and subject indicate high-scoring sequence pairs.

Sequence similarity is performed by BLASTN using default or user specified e-value cut-off. Results are tabulated to indicate high scored hits together with accessions of hosts of subject islands and their descriptions. Hits are hyperlinked to allow users to inspect the subject island contained in Pre_GI and scrutinize BLASTN visualizations for high scored hits. This utility was used for the production of Figure 3 . In this example an island predicted by SWGIS at position 3 609 844–3 653 217 in the genome of *Serratia marcescens* FGI94 [NC_020064] is under consideration. The nucleotide sequence of this island was searched by BLASTN for a high scored alignment through the database. The link ’blastn’ should be used from the home page to open the corresponding input page for the BLASTN alignment. Resulted from this search, various islands from *E. coli* and some other genomes showed significant sequence similarity. The top BLASTN hit for a given newly predicted island is graphically presented in Figure 3 .

### Pre_GI compositional similarity search

Nucleotide sequence of novel islands may be compared against the database entries by OUP similarity searches. Nucleotide sequence of an island is first compared against the 420 cluster/subcluster island representatives in search for shared compositional similarity above 75%. The novel island is then compared with all members of the cluster/subcluster with which similarity was found. The results are tabulated from highest to lowest compositional similarity with subject hits hyperlinked to allow for further inspection. The subject description and clusters/subclusters are displayed together with the percentage similarity. Clusters/subclusters are furthermore hyperlinked to view all islands contained in the specific grouping.

It should be noted that because of the use of this heuristic approach instead of a pairwise comparison of novel islands against the whole database several compositional similarity links may be overlooked.

The computational intensity of a pairwise comparison against the totality of the database would require ample time and as such the implementation of the island representative approach was deemed an appropriate balance between efficiency and time. We persist in working on increasing the speed and accuracy of the compositional search algorithm.

### Filtering of similarity searches

The results of sequence or composition similarity searches may be additionally filtered by specifying a top level taxonomic unit in the field ‘Host lineage’ or metadata on habitat of isolation or growth preferences, e.g. thermophile, anaerobe, in the field ‘Host Information’. The ‘Search’ button applies the filter and the ‘Reset’ button removes filtering.

### Analysis of GenBank files of newly predicted Islands

The ability for users to upload and compare GenBank files of islands produced by SWGIS is of great practical use. Sequence and compositional similarity results are available for up to eight island GenBank files in a single run. Results include the source accessions, source descriptions, island locations and island OU parameters together with sequence and compositional similarity hits to the database as described above. Donor–recipient flux is estimated for user submitted islands. This enables the user to perform multiple comparisons across the database with all of the applications simultaneously. The OUP similarity hit table includes lists of possibly homologous islands stored in Pre_GI supplemented with detected possible donor– recipient relations between them. The resulted web-page allows filtering of records in the table to display only those organisms which may be the donors of the query, or those which are likely recipients of the query islands. The clustering/subclustering identification is of importance to reveal potential ontological links. Web-pages with BLASTN alignments provide the option to visualize high scored BLASTN hits. More case studies of the use of Pre_GI analytical facilities are present in the help/demo web-page available from the home page of Pre_GI ( http://pregi.bi.up.ac.za/index.php ).

### Fragmentation of islands in genomes

**Figure 4.**
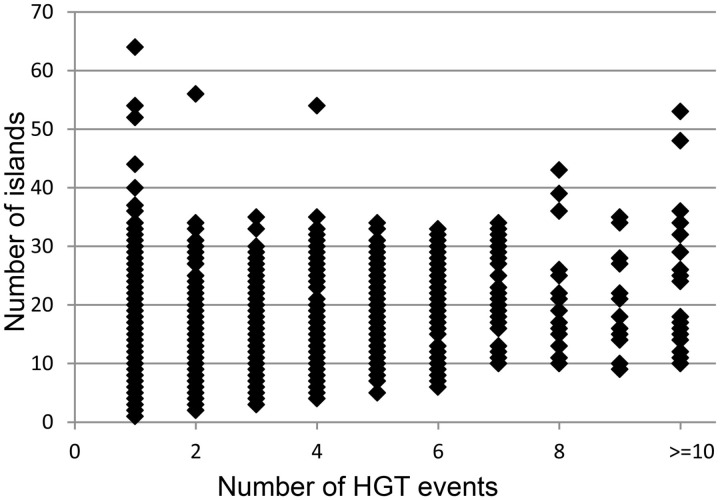
Distribution of number of identified islands and estimated events of HT. Each data point represents a bacterial genome. The vertical axis indicates the number of islands predicted in a genome. The amount of distinct acquisitions of the islands for each genome is depicted on the horizontal axis.

It was hypothesized that acquisitions of islands by genomes are followed in time by fragmentation of the inserts. Pre_GI data may illustrate to which extent this process may perpetuate in different organisms. Based on empirical observations it was assumed that islands resulting from fragmentation of bigger inserts should share significant OUP similarity above 80% and the difference in distance from OUP of islands to the OUP of the host genome should not exceed 15%. Those islands which were too similar to the host OUP (<15% difference) were excluded from consideration as this similarity may result from genomic amelioration that gradually altered all foreign DNA inserts compositionally similar to the sequences of the host organism and eventually similar to each other independently of their origins. With these thresholds it was possible to identify up to 10 groups of islands of different origins per genome as bigger number of groups would overlap. Intermediate islands which showed similarity to representatives of different groups were also ignored. The results of this analysis are summarized in Figure 4 . Each data point represents a bacterial genome with the number of islands predicted in said genome indicated on the vertical axis. The horizontal axis depicts the number of different origins for the islands found in a genome.

### Database statistics and interactions between islands distributed in different bacterial taxa

**Figure 5.**
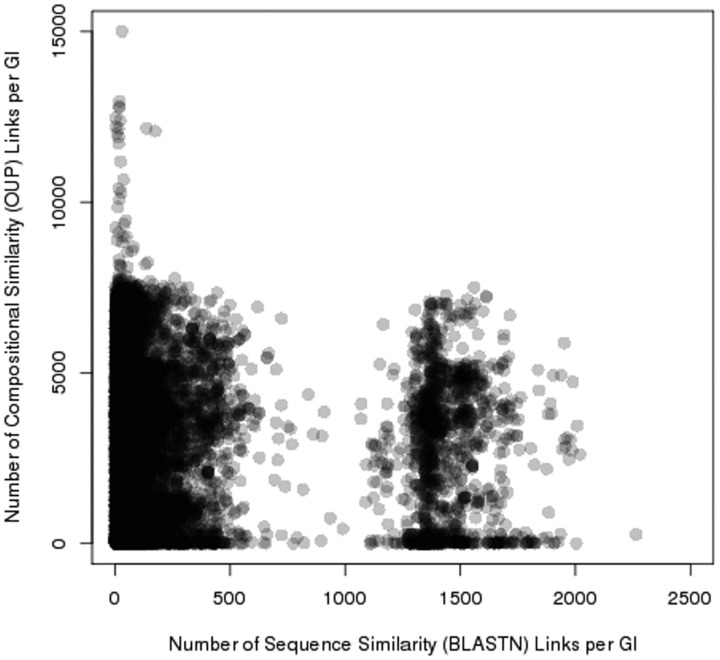
Distribution of sequence similarity (horizontal axis) and composition similarity (vertical axis) links stored in Pre_GI. Each spot represents an island. The congregation of spots (islands) result in darker groupings. Two groups of islands are visible: one rich in BLASTN links, and the other poor of sequence similarity links.

Pre_GI contains 69 176 627 records of OUP similarity links above 75% and 3 692 401 BLASTN similarity records with e-values below 10 ^−^^6^ , i.e. each island in the database in average share similarity with 2586 and sequence similarity with 138 other islands. Two distinct groups where observed by comparing the number of OUP links to the number of BLASTN links for each island ( Figure 5 ). One is characterized by a relatively small number of BLASTN links (in average 150 per island) and islands of the other group have multiple BLASTN links (in average 1500). Islands of the latter group comprise of highly conserved sequences of chromosomal origin: tRNA, ribosomal RNA, ribosomal protein genes and other conserved genes e.g. genes encoding gyrase subunits, etc.

**Figure 6.**
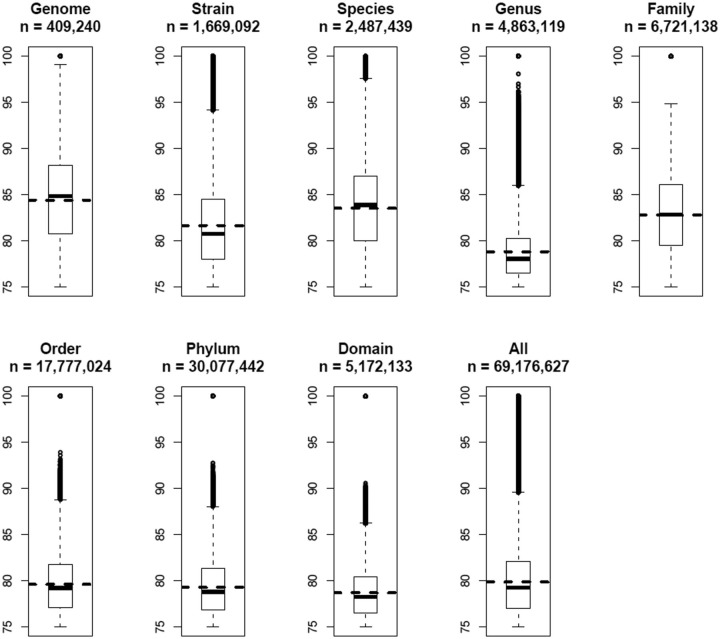
Boxplots of OUP similarities between islands of different taxonomical levels. OUP similarity links were grouped into eight categories: Genome—compositional similarity links between islands found in the same genome; Strain—links found between islands in genomes of two different strains of the same species; Species—links different species of the same genus; Genus—different genera of the same family; Family—different families of the same order; Order—different orders of the same phylum; Phylum—different phyla of the same domain; and Domain—OU links between islands found in bacteria of different domains. Statistical values were also calculated for all 69 176 627 OU links stored in Pre_GI. Numbers of OU links in each category are indicated. Horizontal dashed lines depict the mean value for each category. Vertically adjacent boxes represent lower and upper inter-quartiles with the median values as the line of adjoining. Vertical dashed lines with bars correspondingly represent lower and upper outer-quartiles of distribution. The links above and below the outer-quartiles are shown as small open circles.

All 69 176 627 OUP similarity records were grouped in a way to represent links between islands found within the same genome; between different strains of the same species; between different species of the same genus and so on along the hierarchy of the taxonomic units up to domains. The result is presented in Figure 6 . There is a general trend of diminishing of average values of OUP similarity values between more distantly related organisms and the islands harboured by the same genome showed the highest OUP similarity. Islands found in different strains of the same species are less similar to each other than the islands shared by different species belonging to the same genus. In a similar fashion there is a drop in similarity on the level of genera but an increase of compositional similarity on the level of families.

## Discussion

In this article, we introduce a new database of islands Pre_GI, Version 2015. To exclude confusion and misapplication of the database the concept of ‘island’ relevant to this database is defined as a horizontally acquired fragment of DNA in a bacterial/archaeal genome, including those, which may have been be acquired by ancestral organisms and transferred vertically to its progeny. For example, islands stored in Pre_GI, which were predicted in *Mycobacterium tuberculosis* and *M. bovis,* appear to be ancient inserts acquired by a common ancestor of both these species, as they are known to be extremely clonal and resistant to HT ( 34 , 35 ). It should be stressed that the SWGIS program may identify only relatively large inserts exceeding 5 kb. SWGIS also failed to identify those inserts which initially shared high level compositional similarity with the host genome or acquired a similar OUP to that of the host due to amelioration. Thus we are unable to guarantee that all HT event are represented in Pre_GI.

In general, the number of islands predicted per genome was independent of the eclectic composition of islands. For example, 64 islands found in *Stigmatella aurantiaca* DW4_3_1 were fundamentally uniform in DNA composition and possibly originated by fragmentation of one large insertion. The same may be true for 54 islands of *Clostridium saccharoperbutylacetonicum* N1-4(HMT) and 52 islands of *Clostridium pasteurianum* BC1. Motley islands of at least 10 different origins, which were found in different genomes, are presented in Table 1 . Among bacteria acquiring genes from promiscuous sources there were many symbiotic/pathogenic organisms and extremophyles characterized by unusual enzymatic activities which might result from combinations of genes from different origins.

**Table 1.** Bacterial genomes with eclectic GIs

Species and strain	Phylum	Short description ^a^	No GIs
*Bacteroides fragilis* 638R	Bacteroidetes	Gut microflora, symbiont, pathogen	35
*Bacteroides salanitronis* DSM 18170	Bacteroidetes	Gut microflora, symbiont	39
*Bifidobacterium asteroides* PRL2011	Actinobacteria	Gut microflora, symbiont	19
*Cellvibrio japonicus* Ueda107	Gammaproteobacteria	Soil bacterium	32
*Corynebacterium diphtheriae* 241	Actinobacteria	Pathogen	18
*Corynebacterium diphtheriae* VA01	Actinobacteria	Pathogen	16
*Denitrovibrio acetiphilus* DSM 12809	Deferribacteres	Marine bacterium, biodegradation of pollutants	10
*Desulfitobacterium dichloroeliminans* LMG P-21439	Firmicutes	Soil bacterium, biodegradation of pollutants	14
*Desulfovibrio piezophilus* C1TLV30	Deltaproteobacteria	Deep-sea sulfate reducer	20
*Eubacterium limosum* KIST612	Firmicutes	Carbon monoxide-utilizing acetogen	33
*Fibrella aestuarina* BUZ 2T	Bacteroidetes	Filamentous marine bacterium	28
*Fibrobacter succinogenes* subsp. *succinogenes* S85	Fibrobacteres	Cellulolytic organism	24
*Geobacter uraniireducens* Rf4	Deltaproteobacteria	Uranium bioremediation organism	30
*Granulicella mallensis* MP5ACTX8	Acidobacteria	Tundra soil organism	26
*Hahella chejuensis* KCTC 2396	Gammaproteobacteria	Marine bacterium producing an algicidal agent	57
*Nitrosococcus halophilus* Nc4	Gammaproteobacteria	Marine bacterium	24
*Nitrosococcus oceani* ATCC 19707	Gammaproteobacteria	Marine bacterium	26
*Octadecabacter antarcticus* 307	Alphaproteobacteria	Polar marine bacterium	17
*Octadecabacter arcticus* 238	Alphaproteobacteria	Polar marine bacterium	12
*Paenibacillus mucilaginosus* KNP414	Firmicutes	Soil silicate degrading bacterium	36
*Parabacteroides distasonis* ATCC 8503	Bacteroidetes	Gut microflora, symbionts	45
*Prevotella dentalis* DSM 3688	Bacteroidetes	Oral microflora, symbionts	14
*Pyrobaculum arsenaticum* DSM 13514	Crenarchaeota	Arsenate-reducing hyperthermophile	20
*Pyrobaculum oguniense* TE7	Crenarchaeota	Hyperthermophile	19
*Rothia dentocariosa* ATCC 17931	Actinobacteria	Oral microflora, symbionts	13
*Serratia symbiotica* str. ‘ *Cinara cedri* ’	Gammaproteobacteria	Insect endosymbiont	17
*Spirochaeta africana* DSM 8902	Spirochaetales	Soda lake alkaliphilic anaerobe	12
*Spirosoma linguale* DSM 74	Bacteroidetes	Free-living non-pathogenic bacterium	53
*Teredinibacter turnerae* T7901	Gammaproteobacteria	Endosymbiont of marine shipworms	26

^a^ Short description was taken from corresponding NCBI and GOLDCARD bioprojects

It should be noted that not all genomic fragments with alternative OUP in a given genome were horizontally acquired. SWGIS uses several statistical parameters to minimize false positive predictions ( 14 ) and additionally performs a BLASTN similarity search of the predicted DNA fragments against a database of 16S rRNA sequences to discard candidates containing *rrn* operons.

An assumption was made that islands which share OUP similarity above 75% may have the same origin. It should not be excluded that OUP similarity may be random or due to the amelioration of unrelated islands in the same host genome. Sequence similarity revealed by BLASTN between a pair of islands sharing compositional similarity increases the reliability of the hypothesis of homology between said islands.

It is not unexpected that there are considerably more OUP links between islands than BLASTN hits as it is recognized that frequently islands in a genome are fragments of former larger entities (integrated plasmid, phage or transferred DNA fragment). Thus, these islands share OUP similarity reflecting the common origin but do not necessarily share any homolog genes. Distribution of OUP and sequence similarity links per island formed two distinct groups. The group rich in BLASTN links consisted of highly conserved sequences of chromosomal origin that included tRNA. Genes for tRNA are known as common recombination attachment sites ( *attP* sites) targeted by phage and conjugative plasmid integrases ( 35 ). These genes tend to group together with genes for ribosomal RNA and proteins. They are believed to be resistant to HT but very often are falsely included by SWGIS in islands sequences as the sliding window approach does not allow separating foreign inserts from their *attP* sites. While including these conserved genes in islands may be considered as an error, the sharing of the same attachment sites by different islands is an important indication of homology of these MGE.

The identification of sequence similarity links by BLASTN reinforces the assumption that the composition (OUP) similarity links reflect possible ontological links between islands. Islands linked by multiple OUP hits but displaying no sequence similarity to each other (in Figure 5 , these links are represented by spots in the upper left part of the figure) most likely share non-ontological similarity that may have resulted from a random convergence of OUP of unrelated mobile elements found in taxonomically diverse bacteria.

It might be expected that closely related organisms would more frequently share compositionally similar islands and this was demonstrated in a general global trend in Figure 6 . Contrary to the expectation, there was an undulating local trend between certain taxonomic groups. A possible reason for this may be the method by which organisms were selected for sequencing in research projects. Frequently different strains of the same species are chosen from varying habitats to represent the whole range of genetic diversity. In contrast, researchers studying taxonomic diversity of a specific habitat often employ sequences of related species which co-exist in different conditions yet under pressure of similar limiting factors specific for the chosen habitat. It seems plausible from Figure 6 that sharing of habitats may have a greater influence on the similarity of islands shared by different micro-organisms than the taxonomic relatedness between them ( 36 ). This agrees with Karberg *et al.* ( 37 ) which explained an observed unexpectedly high synonymous codon usage similarity between horizontally acquired genes in taxonomically distant organisms by drawing from a common gene pool termed a ‘supraspecies pangenome’.

Average values of compositional similarities between islands shared on the levels of orders, phyla and domains are similar to that calculated for all the links, but the maximal values above the top quartiles progressively diminish. It is proposed that genetic exchange is possible even between taxonomically distant micro-organisms, but the majority of identified compositional similarity links with values in the range of 75–80% were most likely false predicted ontological relations resulting from randomly acquired compositional similarity. This proposes that when predicting ontological links between islands the value of the compositional similarity threshold should be estimated by accounting for taxonomic distance between the hosts by increasing the threshold for more diverse organisms.

Island-related information and HT recognition will become increasingly more important in drug resistance and pathogenicity research, and in general bacterial/archaeal evolution studies. The ability to identify HT and islands together with additional information on origin of islands and similarity between islands is vital for studies of bacterial/archaeal evolution.

## Funding

This work was supported by a research grant 86941 provided by the National Research Foundation (NRF) of South Africa. Funding for open access charge: A contribution received from the University of Pretoria APC Fund.

*Conflict of interest* *.* None declared.
